# Accidental Insertion of a Broken Needle into the Pterygoid Mandibular Space during Inferior Alveolar Nerve Block: A Case Report

**DOI:** 10.1155/2022/9626612

**Published:** 2022-08-17

**Authors:** Kazuhiro Terada, Kenji Yamagata, Fumihiko Uchida, Satoshi Fukuzawa, Naomi Ishibashi-Kanno, Hiroki Bukawa

**Affiliations:** ^1^Department of Oral and Maxillofacial Surgery, Faculty of Medicine, University of Tsukuba, Tsukuba, Japan; ^2^Department of Oral and Maxillofacial Surgery, Tsukuba Medical Center Hospital, Tsukuba, Japan

## Abstract

The incidence of needle breakage is estimated to be 0.000007%, and most needle breaks occur during inferior alveolar nerve block (IANB) administration and are localized to the pterygomandibular space. Although immediate surgical intervention is recommended for the removal of the fracture needles, intraoperative localization of a broken injection needle fragment can be challenging. Here, we present the case of a 36-year-old woman who underwent dental extraction of the left lower wisdom tooth under local anesthesia at a private office. At that time, a disposable 33G needle was broken and accidentally inserted into the pterygomandibular space during IANB. A broken injection needle was presented at the base of the left coronoid process of the mandible on computed tomography (CT). We successfully removed the broken injection needle using fluoroscopy and assisted endoscopy under general anesthesia.

## 1. Introduction

Local dental anesthesia via injection is a technique that is performed in daily dental practice. A very rare occurrence is a broken injection needle and accidental insertion. The incidence of needle breakage has been estimated to be 0.000007%, and most needle breaks (70%) occur during inferior alveolar nerve block (IANB) administration and are localized to the pterygomandibular space [1]. Needle fracturing is thought to be caused by manual bending of the needle, incorrect choice of needle diameter, or sudden patient movements [2]. One study reported a critical case in which the broken needle migrated toward the skull base into the carotid space and jugular foramen [3]. Although immediate surgical intervention is recommended for the removal of fractured needles, intraoperative localization of a broken needle fragment can be challenging. We report a case of successful removal of a broken injection needle inserted into the pterygomandibular space during IANB using fluoroscopy assisted by endoscopy, and discuss the possible cause, timing of removal, modern modalities of localization, techniques for removal, and preventive measures.

## 2. Case Report

**Figure 1:**
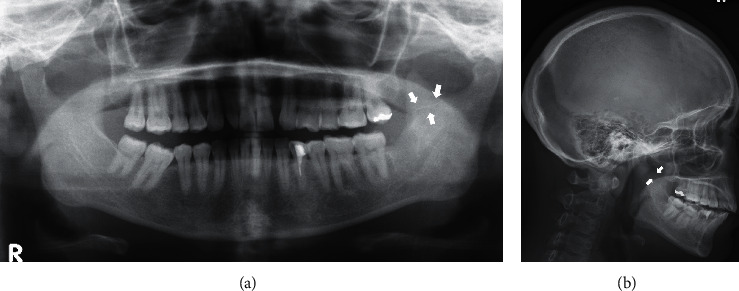
(a) Panoramic X-ray performed at the first visit. The broken injection needle can be seen at the base of the left coronoid process (arrows). (b) Lateral cephalometric X-ray. The broken injection needle can be seen at the base of the left coronoid process (arrows).

**Figure 2:**
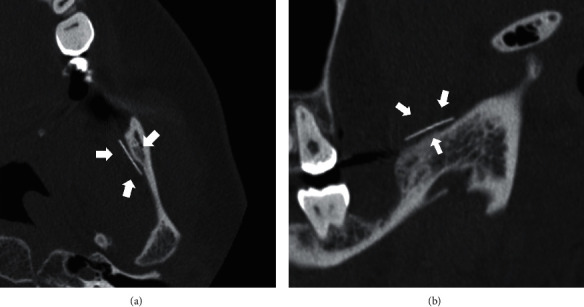
(a) Computed tomography (axial). The broken injection needle can be seen at the base of the left coronoid process (arrows). (b) Computed tomography (sagittal). The broken injection needle can be seen at the base of the left coronoid process (arrows).

A 36-year-old woman was referred to our department for removal of a broken injection needle from the site of a left mandibular third molar extraction. She underwent dental extraction of the left lower third molar under local anesthesia at the private office one month ago. At that time, a disposable 33G needle was broken and accidentally inserted into the pterygomandibular space during IANB. The patient had mild trismus, but no inferior alveolar nerve paralysis, and the extracted site was clearly cured. Mild tenderness was noted in the left submandibular and upper internal deep cervical lymph nodes (LNs). The mucosa on the medial side of the left mandible, which was accidentally inserted with a broken injection needle, was smooth and normal. Panoramic and lateral radiographs revealed the broken injection needle at the base of the left coronoid process (Figure 1(a) and 1(b)). Computed tomography (CT) showed the broken injection needle along the bone surface inside the left coronoid process of the mandible (Figure 2(a) and 2(b)).

**Figure 3:**
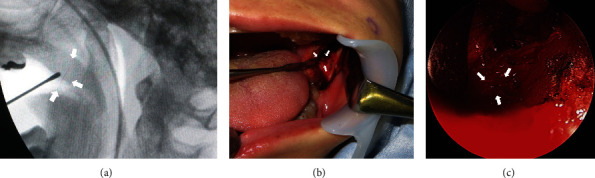
(a) Intraoperative X-ray transmission device images. The position of the broken injection needle was confirmed with a sonde using fluoroscopy. (b) Intraoperative photograph. (c) Endoscopic image taken intraoperatively. An incision approximately 25 mm in length was made on the oblique line of the mandible. A broken injection needle pointing upward from the base of the coronoid process was confirmed in the medial pterygoid muscle. Broken injection needles are shown by an arrow.

**Figure 4:**
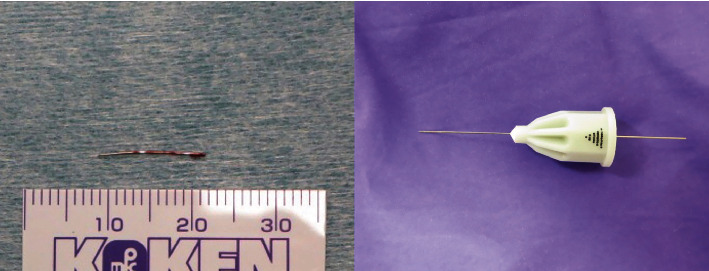
The removed disposable dental injection needle and unused disposable dental injection.

The plan was to remove the broken injection needle via an intraoral approach using fluoroscopy and endoscopy under general anesthesia. First, the position of the broken injection needle was examined using X-ray fluoroscopy (Figure 3(a)). An incision approximately 25 mm in length was made on the oblique line of the mandible. The broken injection needle pointing upward from the base of the coronoid process was confirmed in the medial pterygoid muscle by endoscopic examination (Figure 3(b) and 3(c)). The surgical time was 35 min, and there was a small amount of bleeding. The removed dental injection needle had broken at the base of the hub of the syringe adaptor (Figure 4). The postoperative course was uneventful, with no injury to the inferior alveolar or lingual nerves.

## 3. Discussion

In a meta-analysis, Ho et al. reviewed 78 articles of ocular and neurological adverse effects, allergies, hematomas, needle breakage, tissue necrosis, blanching, jaw ankylosis, osteomyelitis, and isolated atrial fibrillation during dental local anesthesia [4], and estimated that the incidence of needle breakage was around 0.000007%. Fifteen of 16 needle fractures occurred in connection with the IANB, and 13 involved a 30G needle [1]. Acham et al. reported that the diameters of the broken needles were 31G (2.5%), 30G (77.5%), and 27G (20%) [5]. In most cases, needle breakage is not due to a material defect but to preventable reasons such as inappropriate injection techniques or choosing the wrong type of needle [2]. Augello et al. stated that needle fracture is almost always preventable when all precautions are taken. To reduce the risk of needle fracture during dentistry, it is advisable to use a 35-mm-long, 25- or 27-gauge dental needle for administering an IANB [2]. When the broken fragment is not fully buried in the tissues, it can be readily retrieved using a clamp. The patient should be well instructed to avoid sudden head movements before the injection [6]. Our patient was injected with a 33G needle, which is commonly used for pediatric injection. Choosing the wrong type of needle and the injection technique by inserting the base of the needle into the tissue were the reasons for needle breakage.

The timing of surgical removal of the needle fragment was reported to be 73% within 2 days after the incident [2]. In contrast, Acham et al. reported that 53.8% of needle fragments were removed immediately within 1 day after the event, 30.8% within 3 months, and 12.8% were delayed 3–12 months or later [5]. In the present study, the patient was referred to our hospital 1 month after the accident. Fortunately, there was no change in the position of the needle on imaging when she visited our department, and we were able to remove it. In the future, we believe that it will be necessary to inform dental practitioners to consult as soon as possible if a needle is accidentally broken. Brooks et al. reported a critical case of a broken dental anesthetic needle that migrated for several weeks toward the skull base into the carotid space and jugular foramen. They postulated that the broken needle migrated because of the cutting nature of the bevel as well as the musculature of the nasopharynx and oropharynx, specifically the contraction of the superior and middle pharyngeal constrictor muscles [3]. In another case, subcutaneous migration of a broken dental needle from the mandibular gingiva to the neck has been reported. The 33G thick needle migrated to the subcutaneous submandible for 11 months, and was retrieved using an extraoral approach [7]. Therefore, immediate surgical intervention is recommended for fractures of the dental anesthetic needles.

Although the success rate of removal is 95% [5], intraoperative localization of a broken dental needle fragment can be challenging. Conventional CT, intraoperative cone beam CT, sonography, intraoperative navigation, fluoroscopy, metal detectors, and simple plain radiography have all been reported to triangulate broken needles [3]. Recently, Lukas et al. reported successful needle removal using a three-dimensionally printed surgical guide [6]. Real-time intraoperative confirmation of a broken injection needle includes fluoroscopy, endoscopy, and ultrasonography, all of which aid successful removal. In our case, intraoperative fluoroscopy was used to confirm the setting of the incision line and the position of the needle during surgery. The endoscope confirmed the broken injection needle in a bright and magnified image.

Needle breakage prevention is based on three main principles: proper equipment, techniques, and patient care. Most broken needles occur during the administration of IANB. To avoid breakage, long needles, no smaller than 27G, should be used without bending the needle before insertion and without inserting into hub to the tissue. When working with anxious patients, clinicians should be cognizant of sudden or rapid movements [3].

## Data Availability

Data sharing is not applicable to this article as no new data created or analyzed in this study.
